# Monitoring and Assessment of Jump Performance, Workload, and Injury Risk in the Romanian Under-16 Women’s National Volleyball Team

**DOI:** 10.7759/cureus.94050

**Published:** 2025-10-07

**Authors:** Nicolae Stanciu, Cristian Graur, Mark Slevin, Cristian Trambitas, Ioan-Bogdan Bacos, Cezara-Ilinca Stanciu, Gabriel Koszorus, Klara Brînzaniuc

**Affiliations:** 1 Doctoral School of Medicine and Pharmacy, George Emil Palade University of Medicine, Pharmacy, Science and Technology of Târgu Mureș, Târgu Mureș, ROU; 2 Anatomy and Embryology, George Emil Palade University of Medicine, Pharmacy, Science, and Technology of Târgu Mureș, Târgu Mureș, ROU; 3 Physical Therapy, George Emil Palade University of Medicine, Pharmacy, Science, and Technology of Târgu Mureș, Târgu Mureș, ROU; 4 Regenerative Medicine Laboratory, Centre for Advanced Medical and Pharmaceutical Research (CCAMF), George Emil Palade University of Medicine, Pharmacy, Science, and Technology of Târgu Mureș, Târgu Mureș, ROU; 5 Plastic and Reconstructive Surgery, George Emil Palade University of Medicine, Pharmacy, Science, and Technology of Târgu Mureș, Târgu Mureș, ROU; 6 Bioinformatics, George Emil Palade University of Medicine, Pharmacy, Science, and Technology of Târgu Mureș, Târgu Mureș, ROU; 7 Infectious Disease, Clinic of Infectious Diseases, Mures County Emergency Hospital, Târgu Mureș, ROU; 8 Public Health and Health Services Management, Mures County Emergency Hospital, Târgu Mureș, ROU

**Keywords:** acute-to-chronic workload ratio, injury risk, neuromuscular performance, training workload, youth volleyball

## Abstract

Background: Volleyball requires frequent jumping, landing, and overhead movements, predisposing athletes to acute and overuse injuries. Monitoring workload and neuromuscular performance is important for optimizing performance and preventing injuries in youth athletes.

Materials and methods: A retrospective observational analysis was performed using prospectively collected data from a training camp of the Romanian Under-16 (U16) women’s national volleyball team. Twenty athletes were included in the study (mean age: 14.25 ± 0.72 years). Data collection consisted of demographic information, injury history, and perceived exertion, assessed with the modified Borg Category-Ratio 10 (CR10) scale. The training workload (TWL) was derived from session ratings of perceived exertion, while the acute-to-chronic workload ratio (ACWR) was calculated across the four weeks preceding the index week. In addition, neuromuscular performance was evaluated using the OptoJump Next system (Microgate S.r.l., Bolzano, Italy). Athletes completed three standardized tests at both the beginning and end of the camp.

Results: Weekly TWL was similar between groups (injured: 3,723.4; non-injured: 3,664.4). However, only 37.5% of injured athletes remained within the ACWR “safe zone” (0.8-1.3) compared to 75.0% of non-injured athletes. OptoJump assessments revealed no substantial performance differences, and by camp exit, previously injured athletes achieved comparable jump height, reactive strength index (RSI), and relative power to their peers.

Conclusions: Although workloads were broadly similar, injured athletes demonstrated greater fluctuations in ACWR, indicating increased reinjury risk. Effective and personalized rehabilitation allows recovery of neuromuscular performance, but careful workload management remains essential. Integrating load monitoring with neuromuscular assessment may improve injury prevention strategies in youth volleyball players.

## Introduction

In contemporary society, sports fulfill a multi-factorial role. They promote physical health by enhancing cardiorespiratory fitness state, mitochondrial biogenesis, immune regulation, and musculoskeletal resilience [1-3]. They also support mental well-being by reducing anxiety [4,5], improving cognitive function [6,7], and lowering the risk of depression [8,9]. Beyond health, sports contribute to the development of self-discipline, teamwork, and social cohesion, while driving economic growth through tourism and employment opportunities. Moreover, sport serves as a conduit for excellence, innovation, and scientific advancement, underscoring its significance not just for individual health and performance but also within the larger framework of social growth. According to the Fédération Internationale de Volleyball (FIVB), volleyball is classified as one of the top five international sports. The FIVB is considered the world's largest international sporting federation, with 222 affiliated national federations, the governing body responsible for all forms of volleyball on a global level [10]. First introduced in 1895, this sport has one of the greatest participation percentages among any sport in the world. A key feature of volleyball is its accessibility, being played across different age groups, genders, and by individuals with disabilities [11]. Volleyball is classified as a limited-contact discipline and is generally regarded as a safe sport. However, its fast-paced nature and, depending on the athlete’s level of performance, the frequent execution of overhead movements and repetitive jumping expose athletes to both acute and overuse injuries affecting the upper and lower limbs. Acute trauma is particularly common in the net area, where close-range offensive and defensive actions increase the risk of collisions and landings, making injuries to the shoulders, hands, knees, and ankles more prevalent [12]. This sport imposes repeated stress on the shoulder from frequent spiking and serving, elevating the risk of acute and overuse injuries. Despite the relatively high prevalence of shoulder injuries among volleyball players, few prevention programs have been specifically designed for this sport [13]. Due to repetitive stress and high physical demands, volleyball injuries need to be examined in light of hormonal, metabolic, and physiological sex-specific variations, as such variables affect musculoskeletal adaptations, energy availability, and recovery. It is essential to differentiate between male and female athletes in order to precisely identify injury mechanisms and improve preventive measures [14]. Accurate monitoring of neuromuscular performance is fundamental for both performance optimization and injury prevention in sport. The OptoJump photoelectric cell system has been validated as a reliable tool for assessing common jump tests, including squat jump (SJ), countermovement jump (CMJ), and drop jump (DJ), providing accurate measurements of jump height, flight time, and contact time [15,16]. Research demonstrates high validity when compared with force platforms, confirming OptoJump as a practical alternative for field and laboratory settings [17,18]. Although laboratory gold standards such as force plates and three-dimensional motion capture provide more detailed biomechanical data, their cost, limited portability, and specialist requirements restrict routine use in sport environments. In volleyball and related jump tasks, flight-time-based optical systems (e.g., OptoJump, Microgate S.r.l., Bolzano, Italy) show small differences versus force-plate outputs for specific jump types, with some systematic bias depending on protocol and calculation method [19]. This is consistent with reports of strong concurrent validity with force plates [20] and high reliability/validity of optoelectric systems under field-like conditions [21]. At the same time, the literature indicates that complementary measures can enrich interpretation beyond a single modality: pressure-detecting insoles demonstrate concurrent validity and good test-retest reliability for static and dynamic movements [22], while markerless, computer-vision-based 3D motion capture can reach sustainable accuracy for selected kinematic outcomes when camera setup and algorithms are appropriately configured [23]. Accordingly, using OptoJump as a practical primary tool is useful, and, where resources permit, augmenting it with 3D motion capture and validated pressure-insole data can provide a more comprehensive characterization of jump mechanics and athlete loading in applied settings. Moreover, recent studies have shown that OptoJump-derived variables, such as the reactive strength index (RSI), are useful indicators of lower-limb stretch, shortening cycle efficiency, and fatigue resistance, both of which are closely linked to injury risk in athletes exposed to repetitive jumping and landing tasks [15,24]. By enabling coaches and practitioners to track neuromuscular adaptations, asymmetries, and fatigue responses over time, OptoJump facilitates early identification of maladaptive loading patterns, thus supporting evidence-based injury prevention strategies. Given that volleyball is characterized by frequent high-intensity jumping, landing, and explosive movements, integrating OptoJump assessments into athlete monitoring protocols may be particularly valuable for reducing lower-limb injury risk and optimizing training load management in this population.

We aimed to quantify jump performance using standardized OptoJump tests at camp entry and exit (squat jump, 15-second repeated jumps, stiffness hops), monitor internal training load via weekly training workload (TWL) derived from session rating of perceived exertion (s-RPE), compute acute-to-chronic workload ratio (ACWR) across four preceding competition weeks, and examine between-group differences and associations with injury status. Monitoring training load has thus become a cornerstone in injury risk management. The ACWR, which compares short-term fatigue (acute load) against long-term adaptation (chronic load), has been widely studied as a predictor of non-contact injury risk [25,26]. In 2016, the International Olympic Committee (IOC) recommended ACWR as a practical monitoring tool and proposed thresholds for minimizing injury risk [27]. In applied practice, being consistent with IOC guidance, maintaining ACWR within a “safe zone” of 0.8-1.3 is commonly used to guide load decisions and minimize injury risk [25-27]. However, results regarding the correlation between ACWR and injury risk remain inconsistent. Some studies indicate that maintaining values within a moderate range may reduce injury risk [28-30], but others show a linear increase in risk as the ratio rises [31,32]. These conflicting outcomes raised concerns regarding methodological limitations, data biases, and the absence of a clear causal model [33-35]. Nevertheless, predictive models combining ACWR with other workload measures, such as s-RPE, recovery status, and biological maturity, have shown promising results [36-39]. While ACWR alone may not serve as a universal predictor, integrating it with multidimensional monitoring strategies enhances its validity as a practical tool for identifying athletes at increased risk of non-contact injuries. Taking into account the ongoing debate about the predictive utility of ACWR, in this study, ACWR was primarily used as a descriptive monitoring indicator rather than a causal predictor of injury.

The Borg Category-Ratio 10 (CR10) scale has been extensively used as a reliable instrument for quantifying perceived exertion across clinical and exercise contexts [40-41]. Its ratio properties allow for accurate assessment of intensity, with strong associations reported between CR10 scores and physiological markers such as heart rate, blood lactate, and electromyographic activity [42,43]. Building on this foundation, the s-RPE method was introduced, in which the CR10 score is multiplied by session duration to yield a global index of internal training load [44,45]. The ACWR, derived from s-RPE data, has consequently become a practical framework for balancing short-term fatigue against long-term adaptation. Importantly, both the Borg CR10 scale and the s-RPE method are freely available, widely validated, and have been successfully applied in sport-specific contexts to guide training prescription and injury-risk management [46].

## Materials and methods

Study design

A retrospective observational study of prospectively collected data was conducted to assess neuromuscular performance and load responses in Under-16 (U16) female volleyball players from the Romanian national team. The assessments were conducted on the university campus of George Emil Palade University of Medicine, Pharmacy, Science, and Technology (UMFST), Târgu Mureș, Romania, which also served as the designated training facility during the national volleyball team’s training camp.

Informed consent was obtained from all athletes and their legal guardians to undergo the standardized assessments. They did not take part in the design, planning, or implementation of the study. Additionally, athletes and legal representatives acknowledged and agreed that anonymized data from their clinical evaluations could be used for research and scientific publication purposes, with all identifying information removed to ensure confidentiality. The research was conducted in accordance with the principles of Good Clinical Practice (National Institute for Health and Care Research (NIHR)) and the General Medical Council (GMC) guidelines in a private practice environment and received approval from the Ethics Committee of Fizionova Sports Clinic, Târgu Mureș, Romania (protocol no. 85/01.08.2025).

Inclusion criteria

The athletes were selected according to the internal methodology of the Romanian Volleyball Federation, being components of the U16 Romanian national volleyball team.

Data collection

Assessments were performed during the Romanian U16 women’s national team's three-week pre-season training camp in Târgu Mureș (December 2024-January 2025). Neuromuscular performance was measured with OptoJump Next, version 1.13 (Microgate S.r.l.) at two time points: the beginning of the camp and the end of the camp. Training-load history was derived from s-RPE, being calculated as session duration (minutes) x s-RPE (perceived exertion using the Borg CR10 scale) and expressed in arbitrary units (AU). ACWR was calculated as the ratio of the workload in the index week (acute load) to the rolling mean of four preceding competition weeks (chronic load). For injured athletes, the index week was defined as the week in which the injury occurred, and the chronic load comprised the four competition weeks prior to the injury. For non-injured athletes, ACWR was computed over a comparative four-week in-season window matched to the team calendar (W−4 to W−1).

Using the OptoJump Next system, at the beginning and at the end of the camp, athletes performed three standardized tests: 15-second repeated jump test (15-RJ) to evaluate jump height, power, RSI, pace, contact/flight times, and verticality; a stiffness hop test (SH) to quantify leg-spring behavior through RSI, pace, and temporal parameters; and a single SJ test to determine concentric explosive power. These measures, as described in Table 1, provided insight into lower-limb power, stretch-shortening cycle efficiency, fatigue resistance, and coordination.

**Table 1:** Variables derived from the OptoJump Next system, validated against force platforms 15-second (s) Repeated Jumps: Shorter TCont with stable or greater height and power indicates improved rapid force production under stretch–shortening cycle (SSC) constraints; higher reactive strength index (RSI) (Height÷TCont) reflects better SSC efficiency; higher pace (jumps·s⁻¹) with minimal loss of height suggests fatigue resistance across repeated contacts; higher Verticality denotes consistent vertical take-off and reduced lateral drift. TFlight rises as jump height increases. Stiffness Test: Designed to probe leg-spring behavior (fast, low-knee-flexion hops). Lower TExt (push-off extension time) and TCont with maintained or higher height/power imply a stiffer, more elastic tendon–muscle response; higher RSI and pace indicate superior reactive ability at short ground-contact times. Squat Jump (SJ) Test: A concentric-only action (no countermovement). Greater height reflects explosive concentric power; shorter TCont (push-off time) suggests a faster impulse to take off. Because SJ minimizes SSC contribution, gains here are interpreted primarily as improvements in concentric force–velocity characteristics. References: [15,16]

Test	How it is performed	Key variables
15-s repeated jumps test	Athlete performs maximal continuous vertical jumps for 15s, hands on hips (no arm swing).	TCont. (s) = ground contact time; TFlight (s) = flight time; Height (cm) = jump height; Power (W/kg) = relative power; Pace (step/s) = jumps per second; RSI (m/s) = height/contact time; Verticality = jump direction consistency
Stiffness test	Athlete performs fast, stiff vertical hops with minimal knee flexion, maintaining rhythm.	TExt. (s) = push-off extension time; TCont. (s) = contact time; TFlight (s) = flight time; Height (cm) = hop height; Power (W/kg) = relative hop power; Pace (step/s) = hops per second; RSI (m/s) = height/contact time
Squat jump test	Athlete starts in a 90° squat position, holds for ~2 s, then jumps vertically without counter-movement.	TCont. (s) = push-off time; TFlight (s) = flight time; RSI (m/s) = height/contact time

All tests were performed indoors on a level hardwood court in athletes’ regular volleyball shoes. A standardized warm-up (≈8-10 minutes of easy running and dynamic mobility for the lower limbs) preceded testing, followed by two familiarization jumps per task. The testing order was SJ → SH → 15-RJ to minimize fatigue effects on concentric-only performance. For SJ, athletes started at ~90° knee flexion, hands on hips, held the position for ≈2 s, then jumped without countermovement; one recorded trial was taken after familiarization. For SHs and the 15-RJs, athletes kept their hands on their hips and minimized knee flexion for the stiffness task. Recovery between tests was two to three minutes of passive rest; if any attempt was technically invalid (loss of hand-on-hips, obvious stumble), it was repeated after ≥60 seconds.

Jumps were captured using OptoJump Next, positioned and calibrated per manufacturer guidance; the bars were placed parallel on a single, flat surface with continuous capture enabled and hands-on-hips detection protocol (no arm swing). All trials were conducted under consistent lighting and surface conditions within the same session window to limit environmental variability.

The OptoJump hardware is a commercially available device, while the dedicated OptoJump Next software is free of charge and can be downloaded from the manufacturer’s official website [47]. 

We focused on the 15-RJ test because it simultaneously provides height, RSI, and relative power within a single standardized protocol and offers clearer between-group comparisons while avoiding redundancy from overlapping assessments.

Additionally, athletes completed a structured questionnaire that included demographic data, injury history, and perceived exertion using the Borg CR10 scale for up to one month within training sessions. Training load was calculated through the ACWR. The integration of neuromuscular metrics, perceived load, and injury-related data allowed for the exploration of associations between performance characteristics, training demands, and injury occurrence.

s-RPE was assessed using the Borg CR10 scale, a freely available and open-access tool widely validated for subjective workload monitoring [46]. This approach has been successfully applied in volleyball, where the s-RPE method derived from the Borg CR10 has been validated as a practical measure of internal training load [48].

Injury history was obtained using a structured, study-specific questionnaire aligned with the IOC consensus statement for sport injury/illness surveillance [27]. A reportable injury was defined as a physical complaint sustained during volleyball participation that resulted in time lost from training or a match and/or required recovery therapy/medical attention. For each injury, athletes indicated body region, mechanism (non-contact, contact, or overuse), context (general pre-competition/competition/transition phase), time-loss period, context (training/match/other), and whether medical attention was sought. The questionnaire (in the Romanian language) was administered at camp entry under the supervision of medical and coaching personnel to standardize interpretation and ensure completeness; unclear items were clarified on-site. Incomplete/ambiguous responses were coded as missing and excluded casewise from analyses involving that variable.

Statistical analysis

All statistical analyses were conducted using Python (v.3.11, Python Software Foundation, Wilmington, DE) with dedicated libraries (pandas, numpy, matplotlib) and IBM SPSS Statistics software, version 29 (IBM Corp., Armonk, NY). Descriptive statistics were reported as mean ± standard deviation (SD). Between-group comparisons (injured vs. non-injured athletes) were performed using non-parametric bootstrap resampling (5000 iterations) to compute mean differences and 95% confidence intervals (CI). Effect sizes were calculated as Cohen’s d and interpreted as small (0.2), moderate (0.5), or large (0.8). TWL was analyzed for four consecutive weeks (W-4 to W-1), with group means and CI visualized longitudinally. The ACWR was assessed in relation to the “safe zone” interval (0.8-1.3), and group-level proportions of athletes within this range were calculated with a Wilson 95% CI. OptoJump performance variables (jump height, RSI, and relative power) were compared at baseline and post-camp, and improvement (Δ final-initial) was tested between groups using bootstrap resampling. The significance level was set at p < 0.05, with emphasis placed on effect sizes and CI interpretation rather than binary significance testing.

Data management

All collected data were anonymized prior to analysis. Questionnaires and workload data were initially recorded in Microsoft Excel (Microsoft Corporation, Redmond, WA), while neuromuscular performance data were extracted from the OptoJump system. The datasets were merged and cleaned to ensure consistency across athletes and time points. Each participant was assigned a unique identifier to maintain confidentiality and to allow cross-linking of questionnaire, workload, and OptoJump data. Data integrity checks were performed to detect outliers, missing values, and inconsistencies before statistical analysis.

Injury status was evaluated through self-reported data on significant injuries that required training interruption or recovery therapy. Although the questionnaire was study-specific and not derived from a proprietary or licensed scale, its design and implementation adhered to the open-access consensus definitions and methodological standards for injury surveillance outlined by Clarsen et al. [49]. To ensure consistency and accuracy, athletes completed the questionnaire under the supervision of medical and coaching personnel.

Data quality checks for injury data included internal consistency screening (e.g., concordance between time-loss and medical-attention fields) and dual coding by two authors, with discrepancies resolved by consensus.

## Results

The study enrolled a total of 20 female athletes, with an average age of 14.25 ± 0.72 years, all originating from an urban environment. The group presented an average height of 177.6 ± 8.1 cm and a mean body mass of 63.95 ± 9.84 kg. The calculated body mass index (BMI) was 20.2 ± 2.2 kg/m², with most values falling within the normal range for adolescent athletes. Several players exceeded 185 cm in height, reflecting the sport-specific selection trends characteristic of elite volleyball. Overall, the athletes demonstrated relatively homogeneous anthropometric characteristics, consistent with the physical demands and selection criteria of high-performance volleyball at the U16 level. Based on questionnaire data, eight athletes reported a prior injury that required training interruption or recovery therapy, while 12 reported no such history. Participant characteristics of both groups are summarized in Table 2.

**Table 2:** Participant characteristics (analytical subset) The analytical subset comprised 20 athletes (injured n=8, non-injured n=12). Participant characteristics were broadly comparable between groups (age 14.25 years in both; mass 63.12 vs. 64.5 kg), with a modest height difference favoring the injured group (180 vs. 176 cm).

Group	N	Mean age (years)	Mean height (cm)	Mean weight (kg)
Injured	8	14.25	180	63.12
Non-injured	12	14.25	176	64.5

Athletes completed standardized OptoJump assessments at camp entry and exit and provided perceived training-load information for workload calculations. Workload analyses used only cases with complete four-week data immediately preceding the index week (injured n=8; non-injured n=12).

Weekly TWL

Group-level TWL trajectories over the four-week window (W−4 to W−1) did not show consistent between-group differences. The four-week average TWL was 3723.4 for previously injured athletes and 3664.4 for non-injured athletes, yielding a bootstrapped mean difference of +75.0 (injured minus non-injured) with a 95% CI of −1137.3 to +1215.1.

**Figure 1:**
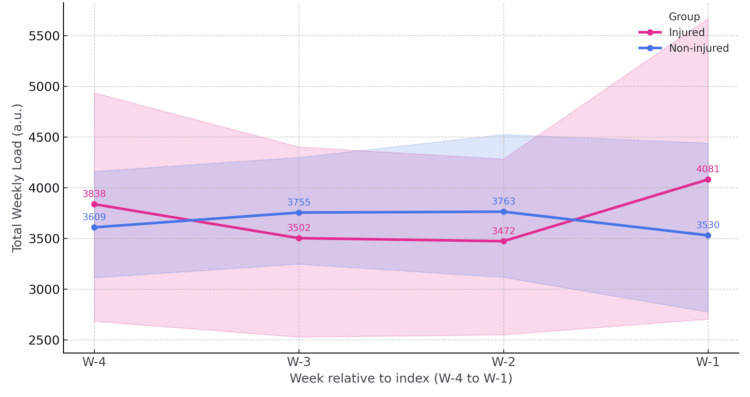
Weekly training workload (TWL): injured vs. non-injured (means ± 95% CI) Group trajectories for injured (red) and non-injured (blue) athletes largely overlap across the four weeks (W). Week-specific differences are trivial–small (Cohen’s d ≈ −0.22 to 0.29); non-injured are slightly higher in W−3/W−2, while injured are slightly higher in W−1, indicating comparable weekly demands overall.

Detailed group means and confidence intervals for each week are presented in Table 3, while the week-by-week bootstrapped differences with corresponding effect sizes are summarized in Table 4. The longitudinal TWL profiles with 95% confidence intervals are displayed in Figure 1.

**Table 3:** Weekly training workload (TWL) summary by group and week (mean, 95% CI, N) Across W−4 to W−1, week-by-week 95% CIs largely overlap between injured and non-injured athletes, indicating similar weekly workloads. Non-injured athletes are slightly higher in W−3/W−2 (3755 and 3763 arbitrary units (AU) vs. 3503 and 3473 AU), whereas injured athletes are higher in W−1 (4081 AU vs. 3530 AU); overall, no consistent between-group difference emerges over the four-week window.

Group	Week	N	Mean TWL	95% CI low	95% CI high
Injured	W-4	8	3837.5	2682.5	4935
Injured	W-3	8	3502.5	2527.5	4402.5
Injured	W-2	8	3472.5	2550	4282.5
Injured	W-1	8	4081.25	2703.72	5667.5
Non-injured	W-4	12	3609.17	3112.4	4160.85
Non-injured	W-3	12	3755	3246.67	4298.33
Non-injured	W-2	12	3763.33	3116.67	4525.04
Non-injured	W-1	12	3530	2775	4440.12

**Table 4:** Between-group differences by week (bootstrap 95% CI, Cohen’s d) Week-by-week differences in TWL (Δ = injured − non-injured) were small, with 95% CIs crossing zero in all weeks and trivial–small effect sizes (d = −0.22 to 0.29). Direction varied across weeks: non-injured slightly higher in W−3/W−2 (Δ −252.5 and −290.83 arbitrary units (AU)), injured slightly higher in W−4/W−1 (Δ +228.33 and +551.25 AU), indicating no clear between-group difference across the four-week window.

Week	Δ Average (injured-non-injured)	95% CI low	95% CI high	Cohen's d	n=injured	n=non-injured
W-4	228.33	-1066.67	1471.12	0.17	8	12
W-3	-252.5	-1378.44	801.71	-0.21	8	12
W-2	-290.83	-1460.9	810.02	-0.22	8	12
W-1	551.25	-1135.03	2331.34	0.29	8	12

ACWR

**Figure 2:**
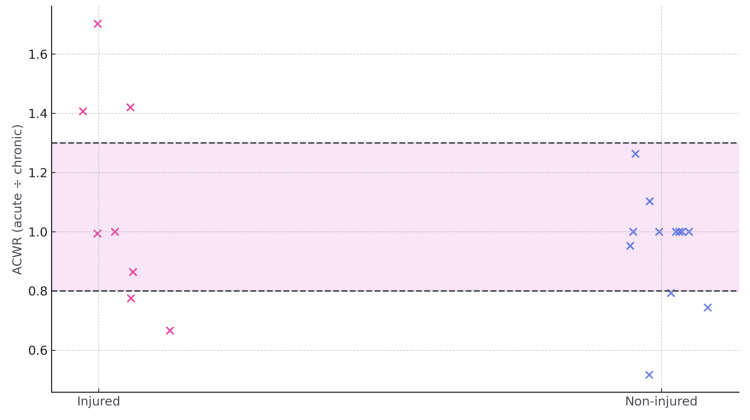
Athlete-level acute-to-chronic workload ratio (ACWR, scatter) with safe zone (0.8–1.3) boundaries defined according to International Olympic Committee (IOC) consensus Scatterplot of athlete-level ACWR values computed for the competition month (acute weekly load divided by the mean load across the four preceding competition weeks), with a shaded band denoting the recommended 0.8–1.3 “safe zone”; red points indicate injured athletes and blue points indicate non-injured athletes, illustrating that a smaller proportion of injured athletes fall within the band and more frequent excursions outside the recommended range). Reference: [27]

The proportion of athletes within the ACWR “safe zone” (0.8-1.3) was lower in the injured group (37.5%, 95% CI: 13.7-69.4; n=8) compared with the non-injured group (75.0%, 95% CI: 46.8-91.1; n=12). Group means were 1.10 for injured athletes and 0.95 for non-injured athletes. The detailed group-level distribution and proportions are presented in Table 5, while individual ACWR values and the safe zone boundaries are visualized in Figure 2.

**Table 5:** Acute-to-chronic workload ratio (ACWR) summary (proportion in safe zone with Wilson 95% CI; group means) Only 37.5% of previously injured athletes (3/8; Wilson 95% CI 13.7–69.4) fell within the recommended 0.8–1.3 “safe zone,” compared with 75.0% of non-injured peers (9/12; 46.8–91.1), and although group means were similar (1.10 vs 0.95), this pattern indicates greater acute-to-chronic load volatility among the injured.

	N	N=safe	Percent=safe	Mean ACWR	95% CI low	95% CI high
Injured	8	3	37.5	1.1	13.7	69.4
Non-injured	12	9	75	0.95	46.8	91.1

OptoJump performance

OptoJump assessments performed at the beginning and end of the training camp showed comparable performance profiles between injured and non-injured athletes. At baseline, group means for jump height, RSI, and relative power did not differ substantially, and by the end of the camp, injured athletes reached performance levels similar to their non-injured peers. Improvements (final-initial) in jump height were positive in both groups, with no statistically significant between-group differences.

**Figure 3:**
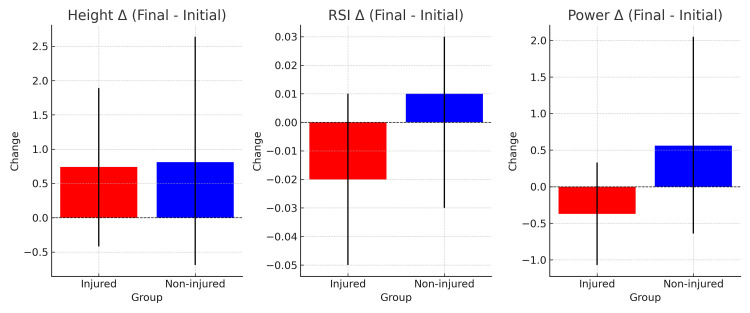
Mean changes (Δ final−initial) in jump height, reactive strength index (RSI), and relative power for injured and non-injured athletes. Bar charts display mean change (Δ final−initial) in (a) jump height (cm), (b) reactive strength index (RSI, m/s), and (c) relative power (W/kg) for injured (red) and non-injured (blue) athletes, with 95% confidence intervals. The dashed horizontal line represents no change. Both groups showed small, overlapping improvements in jump height, with minimal changes in RSI. Relative power tended to decrease slightly among injured athletes while increasing modestly in non-injured athletes. However, the observed changes were modest in magnitude, accompanied by wide variability, and did not indicate a consistent difference between the groups.

Group-level descriptive values for each variable at baseline and post-camp are summarized in Table 6, while the mean changes (Δ final-initial) with corresponding confidence intervals are provided in Table 7. The distribution of improvements in jump height, RSI, and power between groups is illustrated in Figure 3. As noted in the Materials and Methods section, Tables 6-7 and Figure 3 report outcomes from the 15-sec repeated jump test (height, RSI, and relative power).

**Table 6:** Initial and final OptoJump performance (height, reactive strength index (RSI), power). Mean jump height, RSI, and relative power showed broadly comparable values between injured and non-injured athletes at both camp entry and exit. Non-injured players averaged 22.8 ± 1.5 cm at baseline and 23.6 ± 3.0 cm post-camp, while injured athletes averaged 23.6 ± 5.3 cm and 24.4 ± 5.5 cm, respectively. RSI and power values displayed overlapping variability between groups, indicating similar neuromuscular status by the end of the camp.

Group	Height initial (mean ± SD)	Height final (mean ± SD)	RSI initial (mean ± SD)	RSI final (mean ± SD)	Power initial (mean ± SD)	Power final (mean ± SD)
Injured	23.64 ± 5.29	24.38 ± 5.52	0.42 ± 0.11	0.40 ± 0.12	18.77 ± 3.20	18.40 ± 3.69
Non-injured	22.81 ± 1.54	23.62 ± 3.01	0.35 ± 0.06	0.36 ± 0.07	17.14 ± 1.34	17.70 ± 1.90

**Table 7:** Improvement (Δ Final–Initial) with 95% CI, by group (athletes with positive changes included). Among athletes who improved, injured players showed a mean height gain of +1.56 cm (95% CI +0.40 to +2.80), while non-injured players improved by +1.96 cm (95% CI +0.50 to +3.10). Reactive strength index (RSI) gains were modest, averaging +0.03 (injured) and +0.04 (non-injured). Relative power increased slightly in injured athletes (+0.65 W/kg, 95% CI +0.20 to +1.40) and more notably in non-injured athletes (+1.42 W/kg, 95% CI +0.60 to +2.50). Negative or unchanged responses were not included in this analysis.

Variable	Group	Mean Δ	95% CI low	95% CI high	N
Height improvement	Injured	+1.56	+0.40	+2.80	3
Height improvement	Non-injured	+1.96	+0.50	+3.10	6
RSI improvement	Injured	+0.03	+0.01	+0.06	2
RSI improvement	Non-injured	+0.04	+0.01	+0.08	5
Power improvement	Injured	+0.65	+0.20	+1.40	2
Power improvement	Non-injured	+1.42	+0.60	+2.50	5

## Discussion

The present study investigated workload and neuromuscular performance profiles in elite U16 female volleyball players, comparing those with and without a history of injury. The analysis showed no consistent differences in weekly TWL between groups, suggesting that average training demands during the four weeks preceding the index week were broadly comparable. However, athletes with a history of injury were more frequently outside the recommended ACWR “safe zone,” indicating potential fluctuations in acute-to-chronic load balance that could predispose them to injury risk. In line with current debate, we treat ACWR as a surveillance indicator to contextualize recent load history, not as a stand-alone causal predictor of injury.

These findings highlight the importance of monitoring not only absolute workload values but also the dynamic relationship between acute and chronic training stress. Given the observational, single-team design, we interpret these findings as associations rather than causal effects.

Previous research done by Andrade et al. [50] and Gabbett et al. [51] has emphasized the role of workload monitoring in injury prevention across team sports and reported that maintaining ACWR within the 0.8-1.3 range is associated with lower injury incidence, while values exceeding 1.5 increased the risk substantially. Our results are aligned with this evidence, as previously injured players in our cohort displayed higher variability and less time within the safe zone compared with their non-injured teammates. This underlines the need for individualized load management strategies, especially in youth athletes who may experience growth-related vulnerabilities.

OptoJump assessments indicated that previously injured athletes recovered neuromuscular performance to levels comparable with their non-injured peers, as reflected in similar jump height, reactive strength index, and relative power at camp exit. These results suggest that individualized rehabilitation and conditioning programs were effective in restoring neuromuscular function. This is consistent with evidence that integrative neuromuscular training (INT), which combines strength, plyometric, and proprioceptive elements, can correct neuromuscular deficits and reduce reinjury risk in youth athletes [52].

While OptoJump is field-validated, the absence of concurrent force-plate or 3D motion-capture recordings in this cohort means neuromuscular findings should be interpreted with appropriate caution relative to laboratory gold standards.

However, restoration of performance capacity alone does not eliminate injury susceptibility. As highlighted by Jarning et al. [53], excessive or poorly controlled jump frequency remains a major contributor to overuse conditions such as patellar tendinopathy. Therefore, integrating neuromuscular rehabilitation with systematic workload and jump-load monitoring may represent a more effective strategy to ensure both safe return to play and long-term injury prevention in volleyball and other jumping-dominant sports.

Thus, although load imbalance may have contributed to injury occurrence, adequate recovery and training adaptation mitigated long-term performance deficits.

Limitations

This study has several limitations that should be acknowledged. Given the sample size, the study should be interpreted as exploratory, with restricted statistical power and limited representativeness. However, it is important to note that the cohort included the entire Romanian U16 Women’s National Volleyball Team, representing the country’s elite athletes at this age level. Although a larger cohort cannot be obtained because of the national federation regulations, the findings offer significant insights into a distinctive, high-performance group, with generalizability being limited to elite U16 female volleyball in a pre-season camp context.

Data collection was limited to a single national team camp, which does not fully capture seasonal variations in workload, performance adaptations, and injury risk. Monitoring athletes across an entire season, or even multiple years, would offer a more comprehensive picture of long-term load management and injury mechanisms.

Workload assessment was based primarily on internal measures, using the ACWR calculated from s-RPE. This reliance on subjective perception can be influenced by individual differences and recall bias. To mitigate this, standardized questionnaires were used, and athletes completed them under the supervision of coaching and medical staff to ensure accuracy and consistency. Although objective external load markers such as Global Positioning System (GPS) or accelerometry, force-plate, or 3D motion-capture recordings were not available, the use of ACWR remains justified. In 2016, the IOC recommended ACWR as a practical monitoring tool, proposing specific thresholds for minimizing injury risk. Given the current debate about ACWR’s predictive value, we treat ACWR as a surveillance heuristic to contextualize recent load history rather than as a stand-alone predictor of injury risk, and we refrain from causal inference.

Although our injury instrument was study-specific and not formally psychometrically validated, we anchored definitions to IOC consensus guidance and implemented standardized administration and coding procedures, with missing data handled conservatively.

The study’s limitations also highlight the need for future research with larger and more diverse cohorts, objective external load-tracking tools, and longitudinal designs spanning full competitive seasons. Beyond the methods used here, the inclusion of additional functional field-feasible tools (e.g., Y-Balance Test [54,55], Landing Error Scoring System [56], single-leg hop tests [57], Dynamic Postural Stability Index [58], and pressure insoles [22]) alongside laboratory gold-standard technologies such as force plates [59], external load tracking as inertial measurement units (IMUs) [60], GPS/accelerometers [61], and advanced 3D computer-vision-based systems [23] could further refine workload quantification and injury-risk profiling, evaluate whether observed associations persist, and test individualized load-response models.

Such methodological advances would not only strengthen the predictive value of combined workload and neuromuscular performance monitoring but also increase the precision of workload evaluation and enhance the effectiveness of injury-prevention strategies in volleyball and other team sports.

## Conclusions

This study provides descriptive insights into the relationship between training workload, jump performance, and injury history in elite U16 female volleyball players. In this cohort, we observed that weekly training workloads during the four weeks preceding the index week were broadly similar, with week-to-week fluctuations but no consistent group differences between injured and non-injured athletes. However, athletes with a history of injury spent less time within the recommended ACWR “safe zone”, suggesting that fluctuations in load balance may be associated with injury susceptibility. At the same time, OptoJump testing demonstrated that previously injured athletes reached performance levels comparable to their peers, which is consistent with individualized rehabilitation and conditioning programs being effective in restoring neuromuscular function.

Collectively, these findings underscore the value of integrating workload monitoring with neuromuscular assessments to better manage injury risk in youth volleyball. From a practical perspective, individualized recovery strategies and careful load management should be regarded as essential components of both training and rehabilitation protocols. Conclusions are framed conservatively to reflect the study’s observational, single-team design and measurement context.
