# Correction: Monitoring and Assessment of Jump Performance, Workload, and Injury Risk in the Romanian Under-16 Women’s National Volleyball Team

**DOI:** 10.7759/cureus.c367

**Published:** 2025-10-17

**Authors:** Nicolae Stanciu, Cristian Graur, Mark Slevin, Cristian Trambitas, Ioan-Bogdan Bacos, Cezara-Ilinca Stanciu, Gabriel Koszorus, Klara Brînzaniuc

**Affiliations:** 1 Doctoral School of Medicine and Pharmacy, George Emil Palade University of Medicine, Pharmacy, Science and Technology of Târgu Mureș, Târgu Mureș, ROU; 2 Anatomy and Embryology, George Emil Palade University of Medicine, Pharmacy, Science, and Technology of Târgu Mureș, Târgu Mureș, ROU; 3 Physical Therapy, George Emil Palade University of Medicine, Pharmacy, Science, and Technology of Târgu Mureș, Târgu Mureș, ROU; 4 Regenerative Medicine Laboratory, Centre for Advanced Medical and Pharmaceutical Research (CCAMF), George Emil Palade University of Medicine, Pharmacy, Science, and Technology of Târgu Mureș, Târgu Mureș, ROU; 5 Plastic and Reconstructive Surgery, George Emil Palade University of Medicine, Pharmacy, Science, and Technology of Târgu Mureș, Târgu Mureș, ROU; 6 Bioinformatics, George Emil Palade University of Medicine, Pharmacy, Science, and Technology of Târgu Mureș, Târgu Mureș, ROU; 7 Infectious Disease, Clinic of Infectious Diseases, Mures County Emergency Hospital, Târgu Mureș, ROU; 8 Public Health and Health Services Management, Mures County Emergency Hospital, Târgu Mureș, ROU

This article has been revised to add an affiliation for Nicolae Stanciu. The first affiliation has been added: Doctoral School of Medicine and Pharmacy, George Emil Palade University of Medicine, Pharmacy, Science and Technology of Targu Mures, Targu Mures, Romania.

